# SNARE Protein AoSec22 Orchestrates Mycelial Growth, Vacuole Assembly, Trap Formation, Stress Response, and Secondary Metabolism in *Arthrobotrys oligospora*

**DOI:** 10.3390/jof9010075

**Published:** 2023-01-04

**Authors:** Yingmei Zhu, Duanxu Zhou, Na Bai, Qianqian Liu, Na Zhao, Jinkui Yang

**Affiliations:** 1 State Key Laboratory for Conservation and Utilization of Bio-Resources in Yunnan, Key Laboratory for Southwest Microbial Diversity of the Ministry of Education, School of Life Science, Yunnan University, Kunming 650032, China; 2 College of Forestry and Biotechnology, Zhejiang A&F University, Hangzhou 311300, China

**Keywords:** *Arthrobotrys oligospora*, SNARE protein, conidiation, vacuole assembly, trap formation, secondary metabolism

## Abstract

Soluble N-ethylmaleimide-sensitive factor attachment protein receptors (SNAREs) facilitate intracellular vesicle trafficking and membrane fusion in eukaryotes and play a vital role in fungal growth, development, and pathogenicity. However, the functions of SNAREs are still largely unknown in nematode-trapping fungi. *Arthrobotrys oligospora* is a representative species of nematode-trapping fungi that can produce adhesive networks (traps) for nematode predation. In this study, we characterized AoSec22 in *A. oligospora*, a homolog of the yeast SNARE protein Sec22. Deletion of *Aosec22* resulted in remarkable reductions in mycelial growth, the number of nuclei, conidia yield, and trap formation, especially for traps that failed to develop mature three-dimensional networks. Further, absence of *Aosec22* impaired fatty acid utilization, autophagy, and stress tolerance; in addition, the vacuoles became small and fragmented in the hyphal cells of the ∆*Aosec22* mutant, and large vacuoles failed to form. The reduced sporulation capacity correlated with the transcriptional repression of several sporulation-related genes, and the impaired accumulation of lipid droplets is in line with the transcriptional repression of several genes involved in fatty acid oxidation. Moreover, absence of *Aosec22* remarkably impaired secondary metabolism, resulting in 4717 and 1230 compounds upregulated and downregulated in the ∆*Aosec22* mutant, respectively. Collectively, our data highlighted that the SNARE protein AoSec22 plays a pleiotropic role in mycelial growth and development, vacuole assembly, lipid metabolism, stress response, and secondary metabolism; in particular, it is required for the proper development of traps in *A. oligospora*.

## 1. Introduction

Most of secreted proteins enter the endoplasmic reticulum (ER) lumen in eukaryotes [1]. The ER is the largest organelle in cells and plays a critical role in many cellular processes, including protein synthesis, trafficking of proteins, lipid synthesis, autophagy, cellular metabolism, calcium homeostasis, and detoxification [2,3,4,5]. Protein transport involves the budding and fusion of membrane vesicles between adjacent compartments, leading to the entry of the cargo into transport vesicles [6]. The fusion of vesicles with the target compartment depends on the cooperation between several conserved proteins, including small GTPases and soluble N-ethylmalemide-sensitive factor attachment protein receptors (SNAREs) [7,8]. SNAREs play a vital role in the fusion of biological membranes and share a conserved region of 60–70 amino acid residues in plants, animals, and microorganisms, which is called the SNARE domain and is arranged in heptad repeats [9,10]. SNAREs fall into two categories: v-SNAREs in transport vesicles and t-SNAREs in target membranes [11]. v-SNAREs are located in the membranes of vesicles and t-SNAREs are present on target membranes where they assemble into a trans-SNARE complex, forming a fitted connection between two membranes and helping in the mixing of lipid bilayers, thus allowing membrane fusion [12,13]. SNAREs are reclassified as R-SNAREs (arginine-containing SNAREs) and Q-SNAREs (glutamine-containing SNAREs) based on their conserved domain [14]. SNAREs are a large family of proteins; one R-SNARE interacts with three Q-SNAREs, named after their interacting residues arginine (R) or glutamine (Q), when they transport cargoes [15,16].

Sec22 is an R-SNARE protein that plays diverse roles in eukaryotes [17]. Sec22 localizes in the ER and Golgi and helps in anterograde and retrograde transport [18,19]. Sec22 takes part in vacuole function in both yeast and plants [20]. A loss of *sec22* in plants results in Golgi fragmentation and defects in gametophyte development [21]. In addition, Sec22 family proteins are highly conserved from yeasts to humans. Several *sec22* genes, *sec22A*, *sec22B*, and *sec22C*, were also found in humans. In cultured mammalian cells, Sec22 proteins are also required for ER to Golgi trafficking [22,23]. Recently, the R-SNARE homolog Sec22 has been identified in several plant pathogenic fungi. For example, Sec22 is required for conidiogenesis, cell wall integrity, and host plant infection in *Magnaporthe oryzae* [24]. In *Fusarium graminearum*, the R-SNARE protein FgSec22 and the Q-SNARE protein FgSyn8 function similarly in hyphal growth, conidiation, deoxynivalenol (DON) production, and pathogenicity [13,25]. Deletion of the *sec22* homolog *Vdsec22* in *Verticillium dahlia*e resulted in reduced virulence and perturbed secretion of the extracellular proteins involved in carbohydrate hydrolysis [26]. All of these studies demonstrated that Sec22 was a key regulator of the secretory pathway.

Nematode-trapping (NT) fungi are a group of filamentous species that can produce special mycelial traps to capture and digest nematodes in all ecosystems worldwide [27,28]. *Arthrobotrys oligospora* is a typical NT fungus, widely spread in diverse environments, and hence a model to study interactions between fungi and nematodes [29]. *A. oligospora* produces adhesive networks when stimulated in the presence of nematodes or other inducing factors, indicating a switch from the saprophytic lifestyle to the predacious stage [29,30]. In *A. oligospora*, several signaling proteins involved in the regulation of trap formation and pathogenicity have been identified, including mitogen-activated protein kinase (MAPK) [31], G protein β subunit [32], regulators of G protein signaling [33,34], and small GTPases Rab-7A [35], Ras [36], and the Rho family [37]. Recently, a homolog of the yeast SNARE protein Vam7 was revealed to play an important role in vegetative growth, conidiation, trap formation, and ring cell inflation in the constricting ring-forming NT fungus *Drechslerella dactyloides* [38]. However, the functions of SNAREs are still largely unknown in the lifecycle of *A. oligospora*.

This study sought to characterize AoSec22, homologous to the yeast SNARE protein Sec22 in *A. oligospora*, by multiple analyses of targeted gene disruption mutants. Deletion of the *Aosec22* gene results in decreases in vegetative growth, the number of nuclei, the conidia yield, and trap formation; in particular, the deletion mutant (∆*Aosec22*) failed to develop mature traps. Meanwhile, AoSec22 plays a significant role in resistance to diverse chemical stressors. In addition, AoSec22 contributes to the morphology of lipid droplets, autophagy, and vacuole assembly, and mediates secondary metabolism.

## 2. Materials and Methods

### 2.1. Strains and Culture Conditions

The wild-type (WT) fungus *A. oligosopora* (ATCC24927), purchased from the American Type Culture Collection (ATCC), and the mutant strains constructed in this study were cultured on potato dextrose agar (PDA) at 28 °C in the dark. Liquid TG (1% tryptone and 1% glucose) medium was used to cultivate the fungal mycelia for DNA extraction and protoplast preparation. The *Saccharomyces cerevisiae* FY834 strain (a gift from Dr. K.A. Borkovich, University of California), used as a host to construct recombinant plasmids, was cultured in yeast-extract–peptone–dextrose medium. *Escherichia coli* DH5α strains carrying pRS426 and pCSN44 plasmids (Tsingke, Beijing, China) were grown in Luria–Bertani broth agar supplemented with 50 μg/mL kanamycin or 100 μg/mL ampicillin. *Caenorhabditis elegans* was incubated at 25 °C on oatmeal medium and used to induce trap formation in bioassays [39].

### 2.2. Bioinformatic Analysis of AoSec22

The AoSec22 (AOL_s00076g350) was identified based on the amino acid sequences of orthologous Sec22 from the model fungi *Aspergillus nidulans* and *M. oryzae*. The isoelectric point (pI) and molecular weight (MW) of AoSec22 were calculated with the pI/MW tool (http://www.expasy.ch/tools/pi_tool.html) (accessed 19 August 2022), and functional domains were predicted with the default parameters of InterProScan (accessed 19 August 2022). The similarity of homologs from various fungi was analyzed with the DNAman software package (version 5.2.2, Lynnon Biosoft, San Ramon, CA, USA). A BLAST algorithm was used to search for orthologs of Sec22 in different fungi. The resulting sequences were downloaded from the GenBank database for phylogenetic analysis with MEGA 7.0 software [40].

### 2.3. Targeted Gene Deletion of Aosec22 and Southern Blot Analysis

*Aosec22* was disrupted by homologous recombination. Briefly, the upstream and downstream fragments of the *Aosec22* gene were amplified from *A. oligospora* DNA with paired primers (Table S1). The hygromycin-resistant gene *hph* amplified from the pCSN44 plasmid was used as a selection marker. The pRS426 plasmid was digested with *Eco*RI and *Xho*I, followed by co-transformation of the linearized plasmid and the amplified fragments into *S. cerevisiae* (FY834) by means of electroporation. The recombinant plasmid pRS426-AoSec22-hph was then isolated from the yeast plasmids. The target fragment gene for *Aosec22* disruption was amplified from the recombinant plasmid pRS426-AoSec22-hph using primers AoSec22–5f/AoSec22–3r (Table S1) and was transformed into *A. oligospora* using the protoplast transformation method, as described previously [41,42]. Briefly, the fungus *A. oligospora* was incubated in 100 mL of TG broth on a rotary shaker (160 rpm) at 28 °C for 36 h, then the mycelium was suspended in 10 mL MN solution (0.3 mol/L MgSO_4_, 0.3 mol/L NaCl) containing 10 mg/mL of snailase (Solarbio, China) and cellulase (Solarbio, China) for 3–5 h in a shaking water bath (100 rpm) at 28 °C. The protoplasts were resuspended in 100 μL of MTC buffer (10 mm Tris-HCl, pH 7.5, 10 mM CaCl_2_, 1 M MgSO_4_). For transformation, circa 10^7^ protoplasts in 100 μL MTC were mixed with 10 μg of the *Aosec22* replacement fragment and incubated on ice for 40 min. 1 mL of PTC (10 mM Tris-HCl, pH 7.5, 10 mM CaCl_2_, 20% *w/v* PEG 4000) were added and mixed gently. After incubation at room temperature for 1 h, 200–300 μL of the protoplast mixture was added to 5 mL of PDAS medium (PDA supplemented with 10 g/L molasses and 0.4 M saccharose) and poured into Petri dishes. The putative transformants were selected on PDAS medium containing 200 µg/mL of hygromycin B (Amresco, Solon, OH, USA) [43] and further verified by PCR and Southern blotting, as described previously [41].

### 2.4. Analysis of Vegetative Growth and Conidiation

The WT and mutant strains were incubated for 5-day radial growth on PDA, TYGA, and TG agar plates at 28 °C [41]. The spore yield of each strain was measured from the 15-day-old cultures grown on corn meal yeast extract (CMY) medium, as previously described [44,45]. Freshly harvested conidia and hyphae of WT and mutant strains were stained with 20 μg/mL of cell wall-specific calcofluor white (CFW, Sigma-Aldrich, St. Louis, MO, USA) or nucleus-specific 4′,6-diamidino-2-phenylindole (DAPI, Sigma-Aldrich, USA), and visualized for hyphal morphology under an inverted fluorescence microscope [46,47].

### 2.5. Trap Induction and Bioassay

To induce trap formation, 50 μL suspensions (400 spores per μL) of the conidia collected from 15-day-old cultures were incubated on water agar plates at 28 °C for 3 days. Then, 400 nematodes were introduced to each plate for the induction of trap formation, followed by microscopic observation of trap formation and nematode predation at a 12 h interval [45]. The traps were stained with 20 μg/mL CFW and visualized as aforementioned [47].

### 2.6. Microscopy Image Processing

For the staining of vacuole, the 3- to 5-day-old PDA cultures incubated at 28 °C were stained with 5 μM of 7-amino-4-chloromethylcoumarin (CMAC) (Thermo Fisher, Waltham, MA, USA) for 60 min on ice. After three washes with phosphate buffered saline, the stained hyphae were observed under a fluorescence microscopy [48]. For the staining of lipid droplets (LDs) and autophagosomes, hyphal samples were stained with 10 µg/mL of boron dipyrromethene (BODIPY) dye (Sigma-Aldrich, USA) and 10 µg/mL of monodansylcadaverine (MDC, Sigma-Aldrich, USA), respectively, for 30 min in the dark, followed by microscopic observation of the LDs and autophagosomes [37]. In addition, the ultrastructure of mycelial cells was revealed by transmission electron microscopy (TEM).

### 2.7. Analysis of Fungal Responses to Chemical Stressors

To determine the levels of stress resistance, the fungal strains were incubated on TG plates supplemented with or without (control) different concentrations of chemical stressors, including oxidative agents (H_2_O_2_ and menadione), osmotic agents (NaCl and sorbitol), and cell wall-perturbing agents (SDS and Congo red) at 28 °C for 5 days. Relative growth inhibition (RGI) values of the fungal strains were calculated as previously described [31,39]. Three replicates were analyzed for each treatment and control (TG medium alone).

### 2.8. Reverse Transcription Quantitative PCR (RT-qPCR) Analysis

The WT and mutants were inoculated on PDA plates at 28 °C and mycelia were collected on days 3, 5, and 7. Total RNA was isolated from each of the samples using an RNA extraction kit (Axygen, Jiangsu, China) and reversely transcribed into cDNA with a PrimeScript RT reagent kit (with genomic DNA; TaKaRa, Kusatsu, Japan). Primer pairs for the putative genes related to conidiation and lipid acid metabolism were designed (Table S2) and their transcript levels were assessed in the LightCycler 480 SYBR green I master mix (Applied Biosystems, Darmstadt, Germany). The β-tubulin gene was used as an internal standard. The relative transcript level of each gene was calculated using the threshold cycle (2^−ΔΔCT^) method [41].

### 2.9. Metabolomics Analysis

Three hyphal mass discs (7 mm) of each strain were incubated in PD broth for 7 days at 28 °C and 180 rpm, followed by mass collection through vacuum filtration [44]. The biomass was dried, weighed (WT, 3.14 g; Δ*Aosec22*, 2.89 g), and used to prepare extracts of the same concentration. Ethyl acetate (250 mL) was added to extract the fermentation broth and ultrasonic extraction was performed for 40 min. The fermentation broth was allowed to stand for 12 h and the crude extract was concentrated in ethyl acetate under vacuum [44]. The crude extract was dissolved in 500 µL of analytic-grade methanol and the solution was filtered through a 0.22 µm membrane filter for liquid chromatography–mass spectrometry (LC–MS) analysis. The metabolic profiles of the WT and mutant strains were compared using the Thermo Xcalibur software (Thermo Fisher Scientific). Untargeted metabolomics was performed using Compounds Discoverer 3.0 software (Thermo Fisher Scientific) [49].

### 2.10. Statistical Analysis

All experimental data were presented as the mean ± standard deviation (SD) of at least three replicated measurements. The differences between treatments were statistically evaluated by one-way analysis of variance using Prism 9.0 (GraphPad, San Diego, CA, USA). Differences were considered statistically significant if *p* value < 0.05.

## 3. Results

### 3.1. Sequence and Phylogenetic Analyses of AoSec22

*Aosec22* encodes a protein of 215 amino acid residues with a theoretical MW of 23.5 kDa and a pI of 8.34. AoSec22 contains a conserved VARP interface domain and an R_SNARE_SEC22 domain shared by the SNC1 superfamily (Figure S1A). Meanwhile, AoSec22 shares higher sequence similarities with the orthologs of three other NT fungi (89.77–96.74%). In contrast, it shares moderate similarity (60.19–68.37%) with orthologs from other filamentous fungi, such as *Aspergillus fumigatus* and *F. graminearum*, and it also shares moderate similarity (51.87%) with *S. cerevisiae*. Moreover, a phylogenetic tree of orthologous Sec22 proteins from diverse fungi was constructed (Figure S1B) and the orthologous Sec22 proteins from NT fungi were divided into a branch.

### 3.2. Role of AoSec22 in Mycelial Growth and Nuclear Development

**Figure 1:**
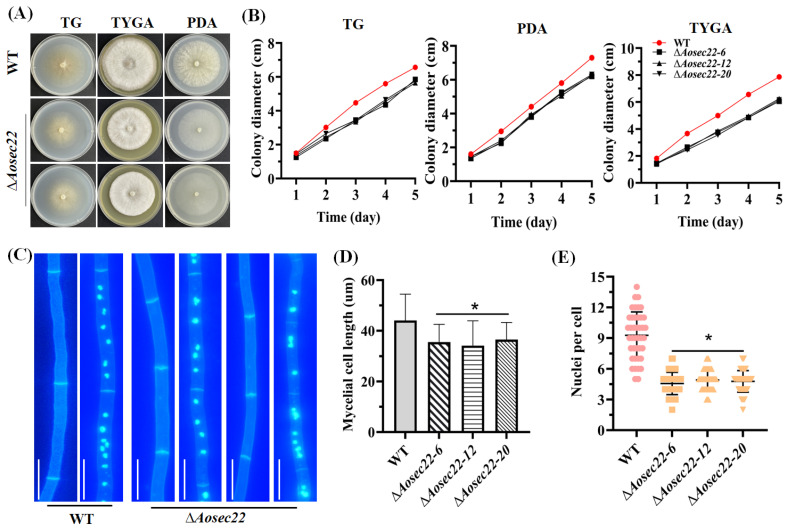
Comparison of mycelial growth of wild-type (WT) and mutant strains in *A. oligospora*. (**A**) Colony morphology of fungal strains incubated for 5 days on PDA, TG, and TYGA plates at 28 °C. (**B**) Diameters of fungal colonies in (**A**). (**C**) Hyphae of the WT and mutant strains stained with calcofluor white (CFW) and 4′,6-diamidino-2-phenylindole (DAPI) after 7-day incubation on CMY. One hundred hyphal cells were randomly selected for the counts of nuclei. Bar: 10 μm. (**D**,**E**) Length of hyphal cells and count of nuclei in (**C**). Measurements represent the average of three independent experiments. The asterisk indicates a significant difference between the mutant and WT strains (*p* < 0.05).

The gene *Aosec22* was disrupted using homologous recombination (Figure S2A,B) and the transformants were verified by PCR and Southern blot procedures (Figure S2C,D); five positive transformants were obtained and three mutants (Δ*Aosec22-6*, Δ*Aosec22-12*, and Δ*Aosec22-20*) were randomly selected for this study. In assays for 5-day radial growth on PDA, TYGA, and TG plates at 28 °C. The growth of the Δ*Aosec22* mutants were remarkably slower than the WT strain (Figure 1A,B). Compared to the WT strain, the ∆*Aosec22* mutants showed not only more septa in hyphae and shorter length in hyphal cells (Figure 1C,D), but also fewer nuclei in hyphal cells of the mutants (3–8 nuclei per cell) versus the WT strain (9–13 nuclei per cell) (Figure 1C,E).

### 3.3. Essentiality of AoSec22 for Conidiation

**Figure 2:**
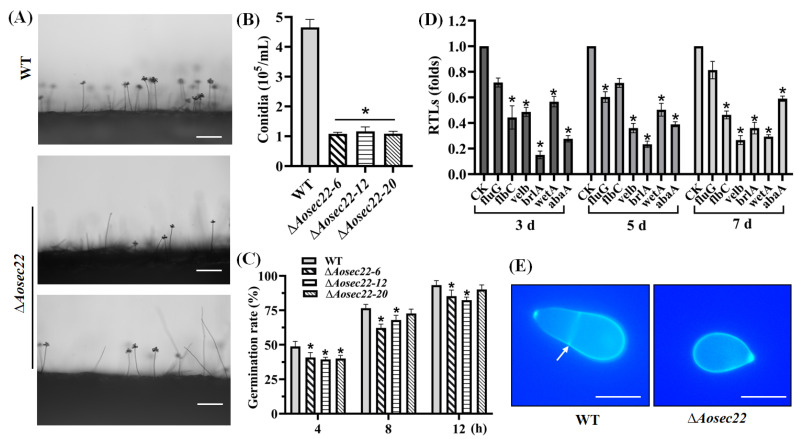
Role of AoSec22 in conidiation and conidial morphology. (**A**) Microscopic images (scale: 50 μm) for conidiation of the WT and mutant strains on PDA. (**B**) Spore yields assessed from 15-days-old cultures. (**C**) Spore germination rate during normal incubation. (**D**) Relative transcription levels (RTLs) of sporulation-related genes in the WT and mutant strains. An asterisk (**B**–**D**) indicates a significant difference between the mutant and WT strains (*p* < 0.05). CK was used as the standard (RTL = 1) for statistical analysis of the RTL of each gene under a given condition. (**E**) Microscopic images (scale: 10 μm) of conidia stained with calcofluor white (CFW). The white arrow indicates the septum.

The disruption of *Aosec22* resulted in reduced conidial production (Figure 2A). The spore yields of the WT and the Δ*Aosec22* mutant strains were estimated to be 4.5 × 10^5^ and 1.5 × 10^5^ spores/mL, respectively (Figure 2B). Moreover, spore germination was slowed down in the ∆*Aosec22* mutants versus the WT strain during normal incubation (Figure 2C). Among six sporulation-related genes analyzed during vegetative growth and sporulation, four (*fluG*, *velB*, *brlA*, and *abaA*) were remarkably downregulated (*p* < 0.05) on day 3 (vegetative growth), especially *brlA*, which was significantly downregulated on days 3, 5, and 7. On the 5th day, four genes (*velB*, *brlA*, *wetA*, and *abaA*) were significantly downregulated, whereas *fluG* and *flbC* did not show a significant difference on the 5th day (Figure 2D). Additionally, four genes (*flbC*, *velB*, *brlA*, and *wetA*) were downregulated on day 7. The WT strain produced an obovoid spore, with one septum formed near the base of the spore; in contrast, 61% of the conidia of the Δ*Aosec22* mutant were morphologically abnormal; these spores lost their septa and became smaller compared with those of the WT strain (Figure 2E).

### 3.4. AoSec22 Is Required for Trap Morphogenesis

**Figure 3:**
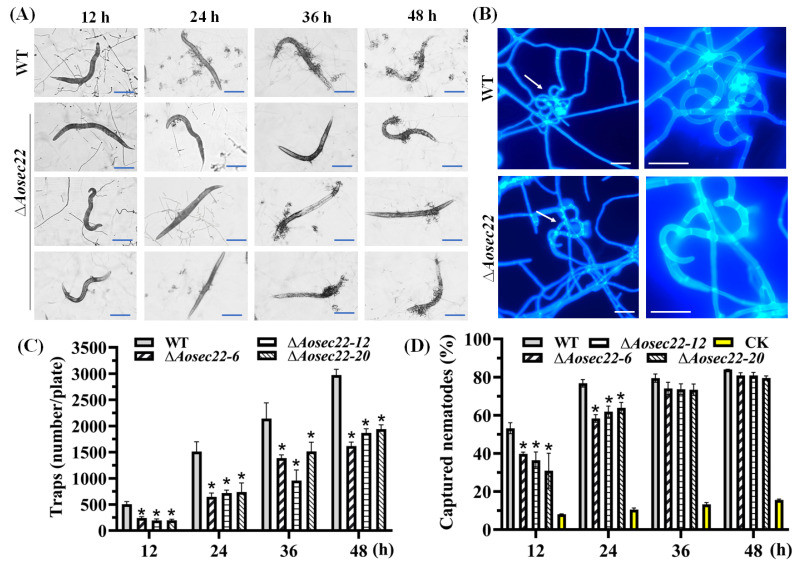
Role of AoSec22 in trap formation, nematocidal activity, and trap morphology. (**A**,**B**) Microscopic images for trap formation and nematode predation at different time points and enlarged traps at 48 h. White arrows: traps. Bar: 100 µm in (**A**) and 10 µm in (**B**). (**C**) Counts of traps produced by WT and ∆*Aosec22* mutant strains at 12, 24, 36, and 48 h. (**D**) Percentages of nematodes captured by WT and ∆*Aosec22* mutant strains at 12, 24, 36, and 48 h. An asterisk (**C**,**D**) indicates a significant difference between the ∆*Aosec22* mutant and the WT strain (Tukey’s HSD, *p* < 0.05).

To examine if AoSec22 is involved in *A. oligospora* trap morphogenesis, we compared phenotypic differences between the WT and the mutants upon exposure to *C. elegans* at different time points. The Δ*Aosec22* mutant formed far fewer traps severely compromised in the development of three-dimensional structures than the WT strain (Figure 3A–C); the traps of the WT strain consisted of 5–8 mycelial loops, contrasting with only 2–3 loops in the mutants’ traps at 48 h post-induction (hpi). Consequently, the number of traps in the Δ*Aosec22* mutant decreased to 35–50% of those in WT (Figure 3C). At 12 and 24 hpi, the Δ*Aosec22* mutant was less capable of capturing nematodes than WT, but its capability was largely restored at 36 and 48 hpi (Figure 3D).

### 3.5. AoSec22 Plays an Important Role in Fatty Acid Utilization

**Figure 4:**
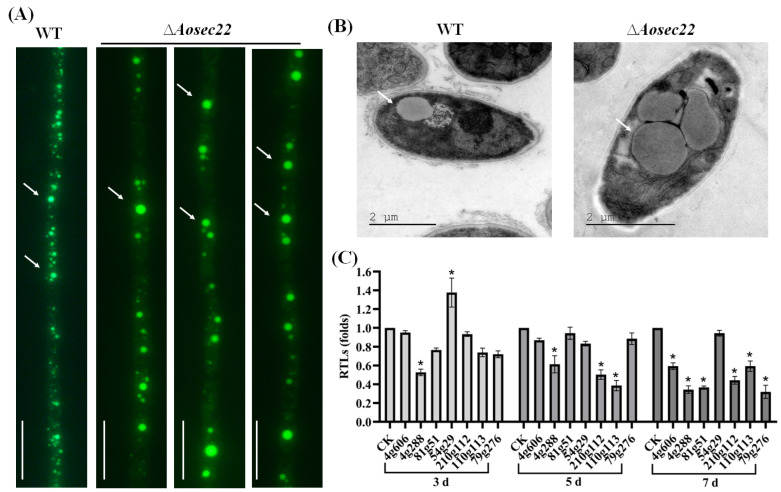
Impact of *Aosec22* disruption on morphology of lipid droplets (LDs) and transcription of genes involved in fatty acid oxidation. (**A**,**B**) Microscopic (scale: 10 µm) and SEM images of LDs in hyphal cells. LDs in (**A**) were stained with 10 µg/mL BODIPY dye. Arrows: LDs. Bar: 10 µm. (**C**) Relative transcript levels (RTLs) of genes related to fatty acid oxidation between ∆*Aosec22* mutant and WT strains on days 3, 5, and 7. CK (standardized to 1) was used as a standard. An asterisk indicates a significant difference between ∆*Aosec22* mutant and WT strains (Tukey’s HSD, *p* < 0.05).

Revealed by hyphal staining and imaging, the mutants’ LDs were enlarged remarkably compared with the WT counterparts (Figure 4A). In addition, large LDs were also present in the ∆*Aosec22* mutants’ TEM images (Figure 4B). To reveal the effect of AoSec22 on fatty acid utilization, seven genes involved in fatty acid oxidation were analyzed, including the coding genes of 3-ketoacyl-CoA ketothiolase (AOL_s00210g122), 3-hydroxybutyryl-CoA dehydrogenase (AOL_s00110g113), acyl-CoA dehydrogenase (AOL_s00079g276), 3-oxoacyl- [acyl-carrier protein] reductase (AOL_s00004g288), phosphatidylinositol transporter (AOL_s00081g51), peroxisomal multi-functional beta-oxidation protein (AOL_s00054g29), and peroxisomal ABC transporter (AOL_s00004g606). As a result, only one gene (AOL_s00054g29) was upregulated in the mutant on day 3, while three and six genes were downregulated in the ∆*Aosec22* mutant on days 5 and 7, respectively (Figure 4C).

### 3.6. AoSec22 Regulates Autophagy and Vacuole Assembly

**Figure 5:**
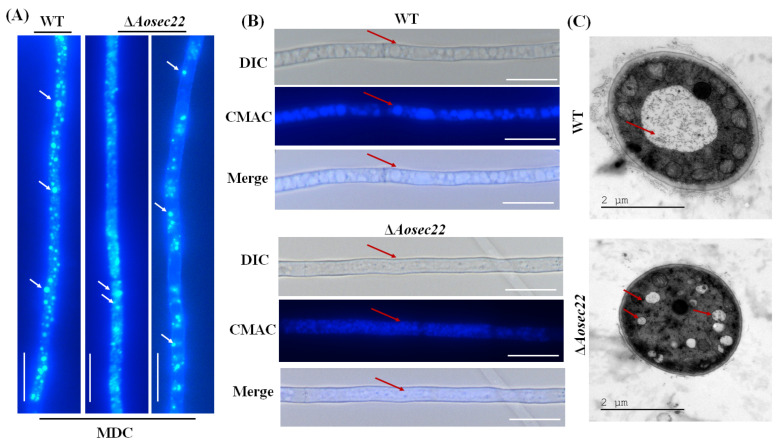
Impact of *Aosec22* disruption on autophagy and vacuolar assembly. (**A**) Microscopic images (scale: 10 µm) of autophagosomes (arrowed) in hyphal cells stained with MDC. (**B**) Microscopic images (scale: 10 µm) of vacuoles in hyphal cells stained with CMAC. (**C**) TEM images of vacuoles in ultrathin sections of hyphal cells. Red arrows: vacuole.

Stained with MDC, autophagosomes were clearly observed in the hyphae of the WT and the ∆*Aosec22* mutant. The WT mycelia contained many autophagosomes distributed in a punctate pattern, whereas far fewer autophagosomes appeared in the hyphal cells of the ∆*Aosec22* mutant and distributed in a dispersed pattern (Figure 5A). To reveal the effect of AoSec22 on vacuole assembly, we stained the WT and mutant hyphae with CMAC, a fluorescent dye specific to vacuoles [48]. The vacuoles of ∆*Aosec22* were small and fragmented, whereas the vacuoles of the WT were regular and large (Figure 5B). TEM images also demonstrated more vacuoles in the hyphal cells of ∆*Aosec22* than of WT (Figure 5C).

### 3.7. Role of AoSec22 in Stress Response

**Figure 6:**
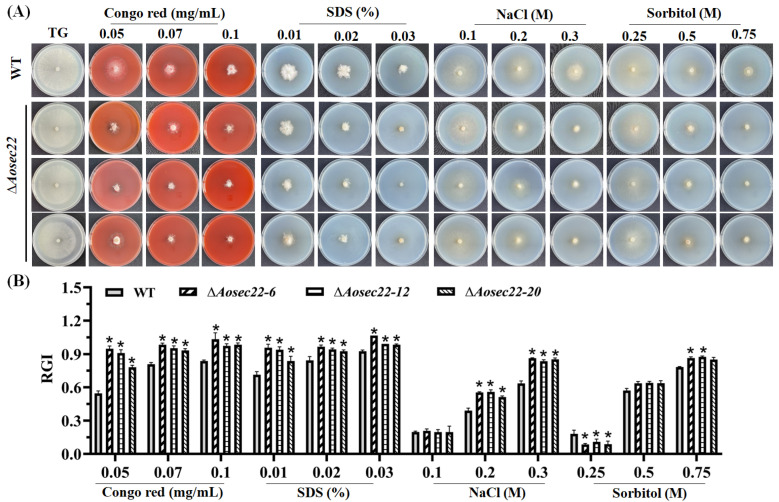
Comparison of stress responses between WT and ∆*Aosec22* strains. (**A**) Colonial morphology of fungal strains under osmotic and cell wall-perturbing stress. (**B**) Relative growth inhibition (RGI) of fungal colonies after 6-day incubation at 28 °C on TG plates supplemented with indicated concentrations of Congo red, SDS, NaCl, and sorbitol. An asterisk indicates a significant difference between ∆*Aosec22* mutant and the WT strain (Tukey’s HSD, *p* < 0.05).

In stress assays, the mycelial growth of the Δ*Aosec22* mutant was suppressed in the presence of cell wall-perturbing agents (Congo red and SDS), and its RGI values were significantly increased (*p* < 0.05) by Congo red (0.05–0.1 mg/mL) and SDS (0.1–0.3%) (Figure 5A,B). Meanwhile, the Δ*Aosec22* mutant also showed increased sensitivity to 0.2 and 0.3 M NaCl and 0.75 M sorbitol (Figure 6A,B). However, H_2_O_2_ and menadione had no influence on the mycelial growth of ∆*Aosec22* mutant, except under 0.75 mM menadione, when the RGI was increased significantly (Figure S3).

### 3.8. AoSec22 Is Involved in the Regulation of Secondary Metabolism

**Figure 7:**
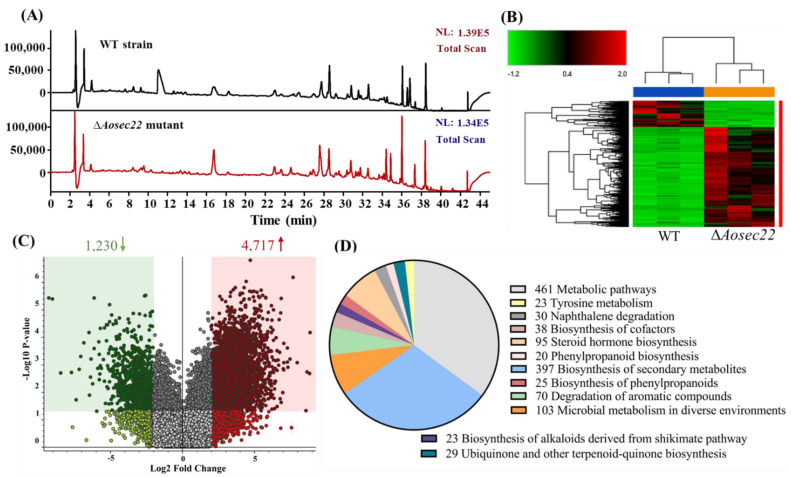
Comparison of metabolic profiling between WT and ∆*Aosec22* mutant strains. (**A**) Comparison of high-performance liquid chromatography profiles of the WT and ∆*Aosec22* mutant strains. (**B**) Heatmap for upregulated and downregulated metabolic pathways between ∆*Aosec22* mutant and WT strains determined via KEGG enrichment. (**C**) Volcano plot of differential metabolites between ∆*Aosec22* mutant and WT strains. (**D**) The number of downregulated and upregulated KEGG pathways in Δ*Aosec22* mutant.

The mycelia of the Δ*Aosec22* mutant turned red after 10-day incubation on PDA (Figure S4A). In addition, LC–MS analysis revealed the difference in the mass and number of compounds in the extracts of the WT and mutant cultures. The WT and mutant strains’ chromatographic spectra differed in the peak areas of several compounds (Figure 7A). A cluster heatmap showed that the metabolic profiles of the Δ*Aosec22* mutant were different from those of the WT, and more metabolic pathways were upregulated in the Δ*Aosec22* mutant (Figure 7B). A volcano plot analysis showed that 1230 compounds were downregulated and 4717 compounds were upregulated in the Δ*Aosec22* mutant compared to the WT strain (Figure 7C). The differential metabolic pathways mainly focused on the metabolic pathways, the biosynthesis of secondary metabolites, the degradation of aromatic compounds, steroid hormone biosynthesis, and microbial metabolism in diverse environments (Figure 7D). Moreover, arthrobotrisins, specific metabolites produced by *A. oligospora* and other NT fungi, were detected in the WT and mutant strains (diagnostic fragments of ions at 139, 393, and 429 *m*/*z* under negative ion conditions) [50,51], and their peak areas were remarkably increased in the mutants (Figures S4B,C).

## 4. Discussion

Sec22 is a member of the SNARE family of proteins, and its homologs play a major role in vesicle trafficking and membrane fusion, which are essential processes for normal cellular functions and homeostasis, and have diverse biological roles in different organisms [10,19]. As presented above, AoSec22 has pleiotropic roles in the lifecycle of *A. oligospora*. Its role in vegetative growth, sporulation, stress response, trap formation, lipid metabolism, vacuole assembly, and secondary metabolism is discussed below.

The deletion of *Aosec22* exerted a profound effect on fungal growth and morphology. Aerial mycelia became thinner and sparser, accompanied by increased septa, reduced cell length, and fewer nuclei in each hyphal cell. The Δ*Aosec22* mutant’s conidiation capacity was lowered and its spore morphology was abnormal. Our observations were consistent with previous observations in *M. oryzae* [23] and *F. graminearum* [24]. In *F. graminearum*, FgSec22 was reported to be indispensable for normal conidiation and conidial morphology [24]. In the present study, the reduced conidiation level correlated with transcriptional repression of several asexual development-required genes, particularly *brlA*, *abaA*, and *wetA*, crucial for the conidiation of *Beauveria bassiana*, *Monascus ruber*, and other fungi [52,53,54]. In *B. bassiana*, loss-of-function mutations of *wetA* have compromised conidiation capacity by 98% [55]. Moreover, BrlA and AbaA are highly conserved activators of asexual development in filamentous fungi [56,57]. BrlA and AbaA are indispensable for aerial conidiation in *Metarhizium roberstii* as well as in *B. bassiana* [52,54]. These results indicate that Sec22 plays a conserved role in sustaining mycelial radial growth, aerial hyphal development, and conidiation in filamentous fungi, and is also crucial for the development of the hyphal septation and nuclear division in *A. oligospora*.

Previous studies have shown that SNAREs are essential for pathogenicity in several plant pathogenic fungi, such as *M. oryzae* [23], *F. graminearum* [24,25], *V. dahliae* [26], and *Colletotrichum fructicola* [58]. In *F. graminearum*, the deletion of *Fgsec22* resulted in reduced pathogenicity [24]. Similarly, CfVam7 is also required for appressorium formation and homotypic vacuole fusion, which are vital for host infection of *C. fructicola* [58]. In the other NT fungus *D. dactyloides*, deletion of *Ddvam7* significantly impaired trap formation and markedly decreased nematode-trapping ability [38]. In our study, the Δ*Aosec22* mutant was capable of forming traps in the presence *C. elegans*, suffered a decrease in the number of formed traps, and hence displayed a defect in developing proper three-dimensional structures. Therefore, the traps formed by the Δ*Aosec22* mutant had fewer loops, implicating an impaired capability of capturing nematodes. These present and previous studies indicate SNAREs are required not only for the pathogenicity of phytopathogenic fungi but also for the development of traps in *D. dactyloides* and *A. oligospora*.

Aside from the growth and trap defects under normal culture conditions, the Δ*Aosec22* mutant was hypersensitive to cell wall-perturbing and osmotic stresses. Previously, the Δ*Mosec22* mutant showed increased sensitivity to cell wall stressors and H_2_O_2_ [38]. The Δ*Movam7* mutant exhibited weakened cell wall and membrane, which were coupled with an abnormal distribution of chitins [59]. Sec22 is involved in the regulation of chitin synthesis in *S. cerevisiae* [60]. Chitin is one of the most important carbohydrates in the fungal cell wall, which maintains structural integrity [61,62]. In *F. graminearum*, the Δ*Fgvam7* mutant was insensitive to some salt and osmotic agents, but hypersensitive to other osmotic and cell wall stressors; moreover, the Δ*Fgsec22* mutant showed increased sensitivity to cell wall stressors [24,63]. These findings indicate that SNAREs, such as Sec22 orthologs, are functional in fungal stress responses, especially in cell wall-perturbing and osmotic stresses of *A. oligospora.*

SNAREs regulate autophagosome formation and are involved in exocytosis in yeast; the endosomal Tlg2, Sec22, and Ykt6 interact with the Sso1-Sec9 complex required for normal Atg9 trafficking [64,65]. Sec22 is not directly involved in exocytosis, as it participates in autophagosome–vacuole fusion and the regulation of the tubular network formation of Atg9 [65,66]. In this study, both autophagosomes and LDs were remarkably reduced in the hyphal cells of the ∆*Aosec22* mutant, accompanied by enlarged LDs. The altered LDs correlated with transcriptional repression of several genes involved in fatty acid oxidation, such as 3-ketoacyl-CoA ketothiolase (AOL_s00210g122), 3-hydroxybutyryl-CoA dehydrogenase (AOL_s00110g113), and acyl-CoA dehydrogenase (AOL_s00079g276). In contrast, the transcript of AOL_s00054g29 was upregulated on the third day, whereas its expression showed no obvious change in the Δ*Aosec22* mutant compared to the WT on the fifth and seventh days. Previously, knockout mutations of SNAREs obliterated tubular network formation and resulted in small Atg9-containing vesicles to stop homotypic fusion events [64,67]. Fungal vacuoles are dynamic, as they undergo a continuous balance of fission and fusion to allow changes in shape, size, and number, and are vital for fungal morphogenesis [68,69]. In this study, the absence of *Aosec22* resulted in a defect in vacuole assembly, since the vacuoles became small and fragmented. Similarly, MoVam7 and MoSec22 are involved in maintaining the shapes of vacuoles in *M. oryzae* [23,59]. The Δ*Movam7* mutant displayed more smaller vacuoles, as seen in the Δ*vam7* mutant of *S. cerevisiae* [59,70]. In the Δ*Ddvam7* mutant, smaller vacuoles were present in the hyphae and uninflated ring cells, making it unable to form large vacuoles in the inflated ring cells [38]. Vacuoles are crucial for nutrient transport in fungi and abnormal vacuoles may interfere with the formation of traps [38]. The mechanisms underlying the formation of large vacuoles and the molecules directly involved remain to be further explored. These findings reveal that Sec22 is required for vacuole assembly and is also crucial for LD utilization and autophagosome formation in *A. oligospora*.

SNAREs are also involved in secondary metabolism in filamentous fungi. In *F. graminearum*, DON production was significantly reduced (by 90%) in the Δ*Fgsec22* mutant strain [24]. In the present study, the loss of *Aosec22* caused the change of colony color and a significant increase in metabolites. KEGG analysis revealed that the differential compounds were enriched in metabolism and related processes, such as the biosynthesis of secondary metabolites and the degradation of aromatic compounds. Moreover, arthrobotrisins are a type of polyketide synthase–terpenoid synthase hybrid metabolites produced by *A. oligospora* and other NT fungi, and are known to inhibit hyphal growth and trap formation [50,51,71,72]. In this study, the arthrobotrisin content was evidently increased in the Δ*Aosec22* mutant, coinciding with its reduced growth and trap formation. Thus, AoSec22 may play an important role in the mediation of secondary metabolism in *A. oligospora*.

## 5. Conclusions

Our results indicate that the SNARE protein AoSec22 plays a vital role in mycelial growth, conidiation, stress response, trap formation, and secondary metabolism in *A. oligospora*. Moreover, AoSec22 participates in the regulation of vacuole assembly and LD morphology. These findings expand the knowledge on the biological functions of the SNARE proteins in NT fungi and provides a basis for future studies on molecular mechanisms underlying vacuole assembly and trap development.

## Data Availability

Not applicable.

## Supplementary Materials

The following supporting information can be downloaded at: https://www.mdpi.com/article/10.3390/jof9010075/s1. Figure S1: Multiple sequence alignment and phylogenetic analysis of WT and *∆Aosec22* mutant strains; Figure S2: Knockout and verification of the genes *Aosec22* in *A. oligospora*; Figure S3: Comparison of oxidative stress responses between WT and ∆*Aosec22* mutant strains; Figure S4: Comparison of the color of colony and the content of arthrobotrisins; Table S1: List of primers for gene disruption used in this study; Table S2: Paired primers for RT-qPCR analysis of genes associated with phenotypes such as conidiation and fatty acid oxidation in *A. oligospora*.
