# Supplementary material for: SNARE Protein AoSec22 Orchestrates Mycelial Growth, Vacuole Assembly, Trap Formation, Stress Response, and Secondary Metabolism in Arthrobotrys oligospora
Source: J Fungi (Basel). 2023 Jan 4;9(1):75. doi: 10.3390/jof9010075 (PMC9863257; doi:10.3390/jof9010075)
Supplement: Supplementary file 1 [file jof-09-00075-s001.zip › jof-2133952-supplementary.pdf]

Supporting Information

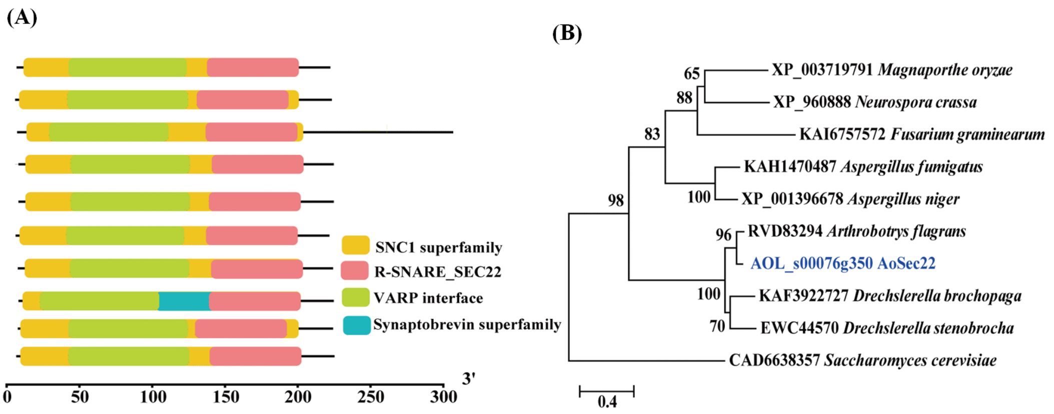

Figure S1. Multiple sequence alignment and phylogenetic analysis of WT and Δ*Aosec22* mutant strains. (A) Prediction of the functional domains of Sec22 proteins. (B) The phylogenetic tree of homologous Sec22 proteins from different fungi.

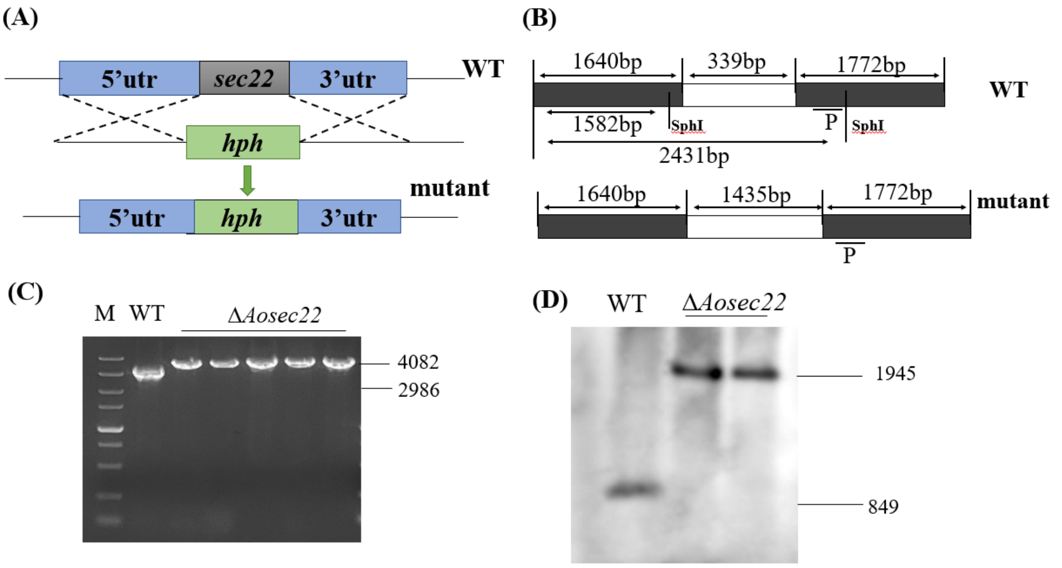

Figure S2. Knockout and verification of the genes *Aosec22* in *A. oligospora*. (A, B) Diagrammatic sketch of homologous recombination of gene *Aosec22*. The homologous flanking sequences of the target gene, Southern blot probe, and the restriction enzyme sites are marked. (C) Positive transformants verified by PCR amplification. M, the DNA marker; WT, the wild-type strain; Δ*Aosec22*, the deletion mutants. (D) The Δ*Aosec22* mutants confirmed by Southern blots.

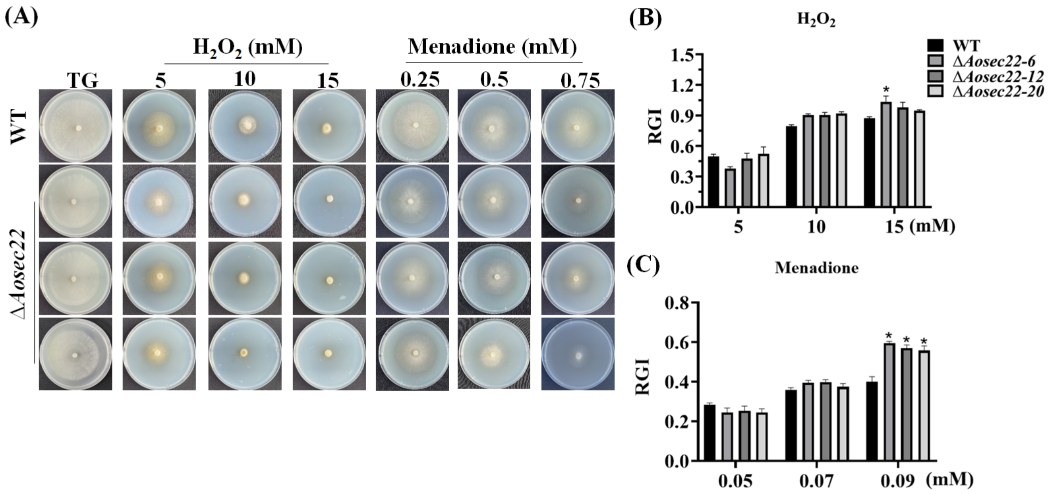

Figure S3. Comparison of oxidative stress responses between WT and Δ*Aosec22* mutant strains. (A) Colonial morphology of fungal strains under oxidative stress. (B) Relative growth inhibition (RGI) of fungal colonies after being grown for 6 days at 28 ℃ on TG plates supplemented with different concentrations of H_2O_2 and menadione. An asterisk indicates a significant difference between Δ*Aosec22* mutant and the WT strain (Tukey's HSD, $p < 0.05$).

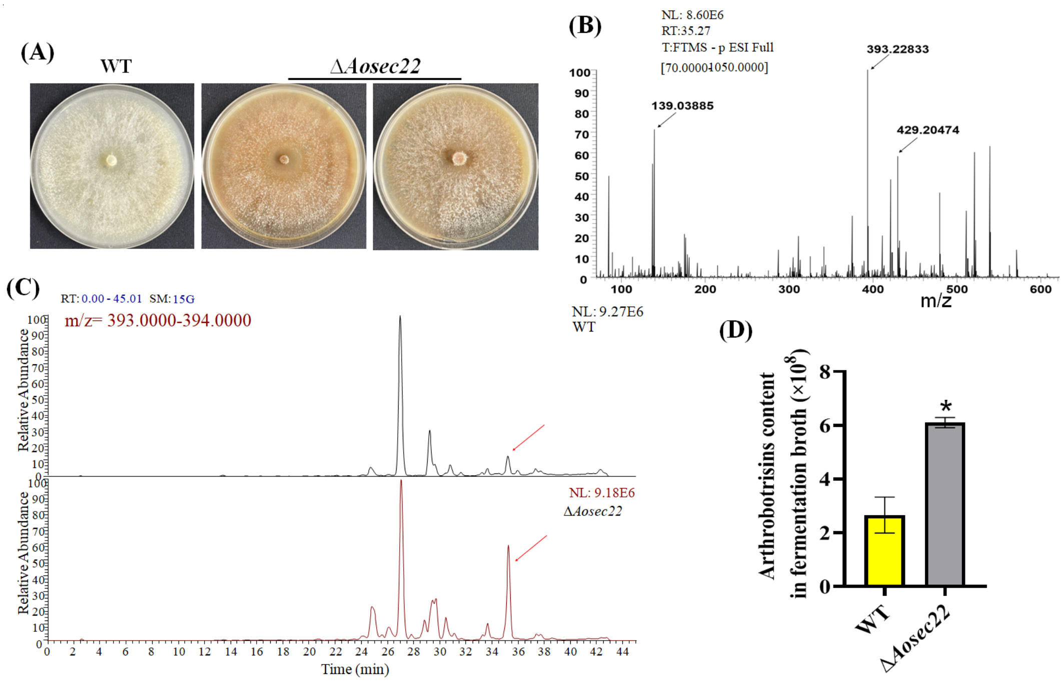

Figure S4. Comparison of the color of colony and the content of arthrobotrisins. (A) The WT and mutant were incubated on PDA medium for 10 days. (B) Mass spectrogram of arthrobotrisins in the WT strain (diagnostic fragments ion at m/z 139, 393, and 429). (C) Mass spectrogram of arthrobotrisins in the WT and Δ*Aosec22* strains (diagnostic fragments ion at m/z 393.0000-394.0000). RT = 35.12 min. (D) The quantification data for arthrobotrisins.

Table S1. List of primers used for gene manipulation in this study.

AoSec22-5F	GTAACGCCAGGGTTTTCCCAGTCACGACGACTCCTGCTGATTGTCCT	Amplify the *AoSec22*
AoSec22-5R	ATCCACTTAACGTTACTGAAATCTCCAACCTTTGTACTGCTGGAGAT	gene 5' flank
AoSec22-3F	CTCCTTCAATATCATCTTCTGTCTCCGACCAAGGGTTATGACGAAGA	Amplify the *AoSec22*
AoSec22-3R	GCGGATAACAATTTCACACAGGAAACAGCTCCGAAGTTTGCGTGTCT	gene 3' flank
Hph-f	GTCGGAGACAGAAGATGATATTGAAGGAGC	Amplify the *hph*
Hph-r	GTTGGAGATTTCAGTAACGTTAAGTGGAT	cassette
YZ-Sec22-F	GCACTCCAGACACCACCCT	Verify the
YZ-Sec22-R	CAGCCACATAACCGTCCC	transformants
Probe-Sec22-F	CAAGGGTTATGACGAAGA	Make Southern
Probe-Sec22-R	CTGAGTCAGATCCGTTCC	blotting probe

Table S2. Paired primers for RT-qPCR analysis of genes associated with phenotypes such as conidiation and fatty acid oxidation in *A. oligospora*.

Description	Gene name	Sequence (5′-3′)
Sporulation-related genes	AOL_s00007g157 (*flbC*)	flbC-5F-CTCTCCGGCAAAGACAATCG
		flbC-3R-GTCGACTGAGGATAGTAGCT
	AOL_s00043g361 (*fluG*)	fluG-5F-GATTCCAGTCCCGTGAATTC
		fluG-3R-GCTAAGGAGAGGATGGGCAT
	AOL_s00080g63 (*abaA*)	abaA-5F-AACTTTATGCGCCTTGTCGT
		abaA-3R-TTGGCTAGGTGGTCTGTACG
	AOL_s00054g811 (*velB*)	velB-5F-ATTCCGCAACTTCTCCCTCA
		velB-3R-GGCATGTTTGGATTCTGGGG
	AOL_s00097g514 (*brlA*)	brlA-5F-AACTCCATCACCATCCGTAA
		brlA-5R-CAGGATATTCGGCACTCA
	AOL_s00173g221 (*wetA*)	wetA-5F-CCCTGTGCTACTATTGCTAC
		wetA-5R-CCGTTGCGAGCATTTCTT
β-tubulin gene	AOL_s00076g640 (*tub*)	tubA-F-CCACCTTCGTCGGTAACTC
		tubA-R-TCGTCCATACCCTCACCAG
Genes related to fatty acid oxidation	AOL_s00004g288	288-5F-AAGAAATCCCACTTCAGAGAGG
		288-3R-TACGTGTCCAGTAACATAGCTC
	AOL_s00081g51	51-5F-GCCGATCCTTACCAAATCATTC
		51-3R-CCAATTCTTTTCCGTAGCTGAG
	AOL_s00210g122	122-5F-GCCGCACATATTGTTAACAGAT
		122-3R-TGATCTTGCTGTTCTCAGTCAT
	AOL_s00110g113	113-5F-CTAACAGAAACTCAAGCATCGG
		113-3R-GGAACCGGATTCATGAAATGAG
	AOL_s00079g276	276-5F-AACAATCCGTCGTTATTGTTCC
		276-3R-GCGATCATGTAGTCTAGTCCTC
	AOL_s00054g29	29-5F-GGTATCTACGGAAATTTTGGCC
		29-3R-GTGCAATATAATCGGGCTTGAG
	AOL_s00004g606	606-5F-TTCGGATTCGTTATTACCTCCC
		606-3R-TAACATGAGTCGCTTGTTTGTG
